# Multi-Parametric Profiling Network Based on Gene Expression and Phenotype Data: A Novel Approach to Developmental Neurotoxicity Testing

**DOI:** 10.3390/ijms13010187

**Published:** 2011-12-23

**Authors:** Reiko Nagano, Hiromi Akanuma, Xian-Yang Qin, Satoshi Imanishi, Hiroyoshi Toyoshiba, Jun Yoshinaga, Seiichiroh Ohsako, Hideko Sone

**Affiliations:** 1 Health Risk Research Section, Research Center for Environmental Risk, National Institute for Environmental Studies, 16-2 Onogawa, Tsukuba 305-8506, Japan; E-Mails: nagano.reiko@tasc-nt.or.jp (R.N.); akanuma.hiromi@nies.go.jp (H.A.); y_qin@envhlth.k.u-tokyo.ac.jp (X.-Y.Q.); Toyoshiba_Hiroyoshi@takeda.co.jp (H.T.); 2 Department of Environmental Studies, Graduate School of Frontier Science, The University of Tokyo, 5-1-5 Kashiwanoha, Kashiwa, Chiba 270-8563, Japan; E-Mail: junyosh@k.u-tokyo.ac.jp; 3 Center for Disease Biology and Integrative Medicine, The University of Tokyo, 7-3-1 Hongo, Bunkyo-ku, Tokyo 113-8654, Japan; E-Mails: imanishi@m.u-tokyo.ac.jp (S.I.); ohsako@m.u-tokyo.ac.jp (S.O.)

**Keywords:** developmental neurotoxicity, embryonic stem cells, high-content screening, Bayesian network modeling, gene expression, multi-parametric analysis

## Abstract

The establishment of more efficient approaches for developmental neurotoxicity testing (DNT) has been an emerging issue for children’s environmental health. Here we describe a systematic approach for DNT using the neuronal differentiation of mouse embryonic stem cells (mESCs) as a model of fetal programming. During embryoid body (EB) formation, mESCs were exposed to 12 chemicals for 24 h and then global gene expression profiling was performed using whole genome microarray analysis. Gene expression signatures for seven kinds of gene sets related to neuronal development and neuronal diseases were selected for further analysis. At the later stages of neuronal cell differentiation from EBs, neuronal phenotypic parameters were determined using a high-content image analyzer. Bayesian network analysis was then performed based on global gene expression and neuronal phenotypic data to generate comprehensive networks with a linkage between early events and later effects. Furthermore, the probability distribution values for the strength of the linkage between parameters in each network was calculated and then used in principal component analysis. The characterization of chemicals according to their neurotoxic potential reveals that the multi-parametric analysis based on phenotype and gene expression profiling during neuronal differentiation of mESCs can provide a useful tool to monitor fetal programming and to predict developmentally neurotoxic compounds.

## 1. Introduction

One of the emerging issues in developmental neurotoxicology is to detect effects of chemicals on fetal programming, which is defined as variations in metabolism, gene expression and genome modification during fetal life that induce or repress the somatic structure and physiological systems after development [1–4]. A significant issue in the prevention of neurodevelopmental deficits of chemical origin is the paucity of testing of chemicals for developmental neurotoxicity [5]. New, precautionary approaches that recognize the unique vulnerability of the developing brain are needed for testing and to control the use of chemicals.

Toxicity testing using embryonic stem cells (ESCs) is an efficient approach for developmental neurotoxicity testing (DNT) [6,7]. Compared with the DNT in animal studies, which are costly, time-consuming, and require considerable numbers of laboratory animals, the ESCs test is unique in that, in a relatively simple cell-line-based assay, it incorporates the entire differentiation route from pluripotent ESCs into differentiated cells [8]. Furthermore, as the neuronal differentiation of ESCs provides insight into the early neurogenesis during embryonic development, several protocols have been developed based on the disturbances of this process to model developmental neurotoxicity [9,10]. A 13-day neural differentiation protocol of mouse embryonic stem cells (mESCs), which is combined with morphological observation, immunocytochemistry, gene expression and flow cytometry, has been applied to assess the developmental neurotoxicity of methyl mercury chloride [9]. More recently, a broad gene expression profile during a 20-day differentiation process of mESCs has been successfully designed, in which transcription-based end points have been used to identify the disturbed neuronal differentiation of mESCs [10]. Developing neurons display plasticity in the type of neurotransmitter phenotype that they can assume, and alterations of synaptic activity and expression of neurotrophic factors can influence the “wiring” of developing neuronal circuits [11]. Consequently, exposure to environmental chemicals that promote or interfere with synaptic activity or expression/function of neurotrophins can result in miswiring, leading to neurobehavioral anomalies. However, a sensitive method for quantitatively measuring alterations of fetal programming during neuronal differentiation, particularly in the connection between the early disturbances and the later outcomes, has not yet been devised.

Here, we produced a high-content and sensitive method for quantitatively measuring the developmental neuronal toxicity of 12 environmental chemicals (see Table 1) using mESCs test combined with DNA microarray analysis, morphological analysis and Bayesian approaches. This confers a new predictive insight for chemical screening in a complex cell culture system that mimics early mammalian embryonic development. We performed multi-parametric profiling of gene expression data sampled at the early stage of mESC differentiation and neuronal phenotype data sampled at a later stage of neuronal cell differentiation after embryoid body (EB) formation. Then, these sampled data were analyzed by a Bayesian network analysis (BNA). This analysis can be depicted graphically to represent the probability structure of the causal complex [12–14].

The Bayesian algorithm used in this study was proposed by Toyoshiba *et al.* as a prediction tool for the effect of exposure to chemicals [15]. The TAO-Gen algorithm is based on the assumption of a linear relationship between changes in the expression levels of two genes following chemical exposure [16], which employs the Gibbs sampling method on the search algorithm to estimate posterior probability distribution [17,18]. The advantage of Gibbs sampling is that it samples from a full conditional distribution and it is an efficient and easy sampling procedure. Gibbs sampling is a Markov chain Monte Carlo method, which involves generating a sample from one or several variables with an acceptance probability of one. This process is repeated until the sampled probability distribution is close to the actual distribution. This algorithm can be used to search for key transcription factors of signal transduction during ES cell differentiate process [19].

**Figure 1:**
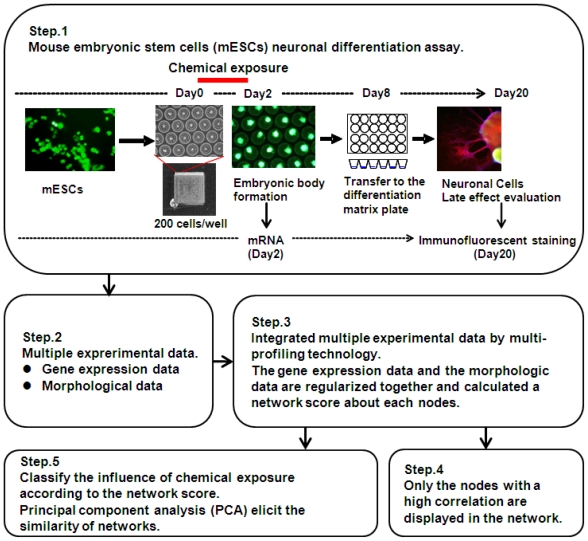
Experimental steps in this study for the assessment of developmental neurotoxicity.

Therefore, the overall aim of this paper is to make a conceptual and methodological proposal to establish a more efficient approach for DNT (Figure 1). More specifically, two objectives are addressed. The first is to describe the DNT design and to identify multi-parametric profiling networks (MPNs) multiple-index networks for 12 environmental chemicals as examples. These are based on the gene expression signatures of mESCs and phenotype profiling of neurons differentiated from EBs. The second objective is to suggest an information-predictive approach to detect alterations of fetal programming that can be made operational using BNA. We propose BNA as an operational tool for empirically applying the DNT approach.

## 2. Results and Discussion

### 2.1. Phenotype Profiling Based on the Morphology of Differentiated Neuronal Cells by High-Content Image Analysis and Generation of Phenotypic Networks

**Figure 2:**
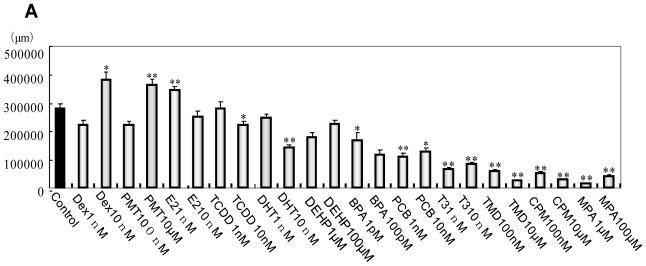

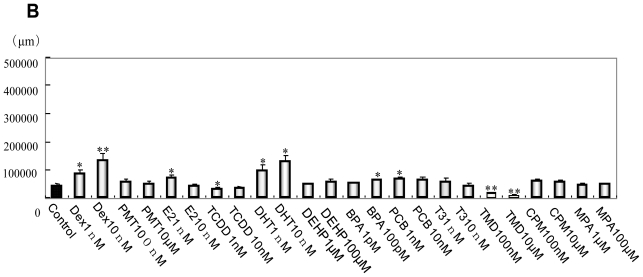
Morphological data of MAP2-positive neurons and glial cells. (**A**) Total length of MAP2-positive neurons per well; (**B**) Total length of glial processes per well. * *P* < 0.05, ** *P* < 0.001 *vs.* the vehicle control (DMSO).

**Figure 3:**
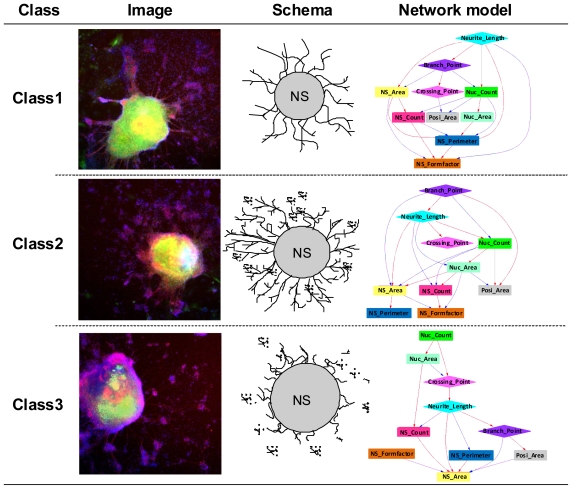
Classification based on morphological imaging and phenotypic feature networks. Class 1: Extension from the turning point is short while the neurite is long; Class 2: Neurite is long and the branch point is complex; Class 3: Neurite is short and there are many nucleus count.

EBs neurally differentiated into neural cells after transfer to OP/L-coated plates. Effects of the 12 environmental chemicals on neural cell growth and NS morphology are shown in Figure 2. Dexamethazone (Dex), Permethrin (PMT) and 17β-estradiol (E2) significantly increased neurite length, while 4-OH-2′,3,3′,4′,5′-pentachlorobephenyl 107 (PCB), triiodotyronine (T3), Thalidmide (TMD), cyclopamine (CPM) and methoprene acid (MPA) significantly decreased neurite length compared with DMSO control (Figure 2A). In glial fibrillary acidic protein (GFAP) positive glial cells, Dex, 5α-dihydrotestosterone (DHT), bisphenol A (BPA) and PCB significantly increased neurite length, while TMD significantly decreased neurite length (Figure 2B). Chemicals were then classified based on morphological features by MPN analysis to extract and predict their toxicities. 12 phenotypic networks (PNs) were generated from the MPN analysis based on the phenotypes of neuronal cells and NSs. We manually classified three categories out of the12 PNs depending on network structures (Figure 3).

### 2.2. Generation of a Comprehensive Network Based on Gene Expression and Phenotype Profiling by a Bayesian Network Model

**Figure 4:**
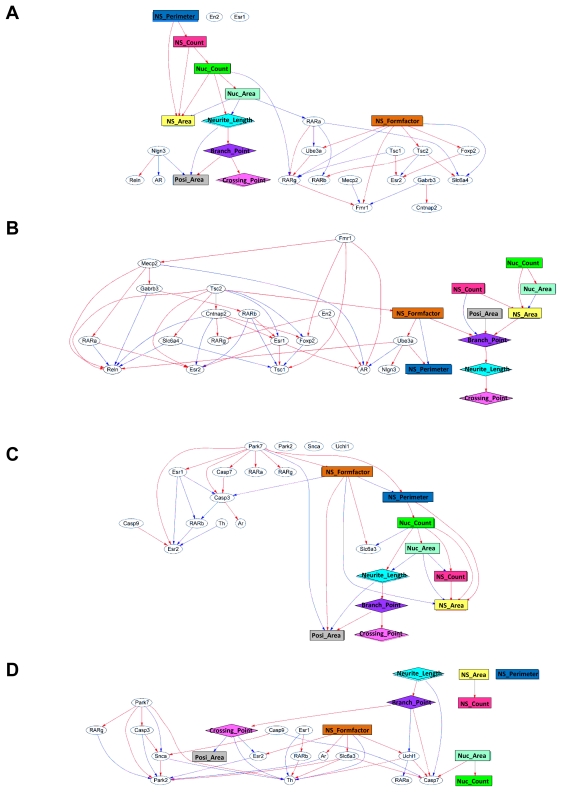
Typical example of GPINs for autism and Parkinson’s disease gene sets. Gene expression and morphological parameters were connected by the strength of the correlation. GPINs of autism related genes and morphological parameters: (**A**) the vehicle control (DMSO) and (**B**) TMD exposure. GPINs of Parkinson’s disease related genes and morphological parameters; (**C**) the vehicle control (DMSO) and (**D**) PMT exposure.

A significant advantage of our unique MPN analysis is that it can predict the correlation coefficient for each pair of nodes, regardless of the data types. Our initial efforts were to derive the interactions between variations of gene expression data after chemical exposure at the early stage of mESC differentiation and effects on the neuronal phenotype data sampled at a later stage of neuronal cell differentiation after EB formation. That is to perform a comprehensive analysis combining data from two different properties. We extracted a discriminative gene group as a gene expression signature from exhaustive genetic profiling, each group was defined by their characteristic category (Table 2) and these gene sets were used in a gene and phenotype interaction network (GPIN) with cell morphological data (Figure 4). To verify whether the MPN analysis can draw out the developmental neurotoxicity, typical examples of DPINs for autism and Parkinson’s disease related gene sets exposed to TMD and PMT, respectively, were discussed.

In DMSO control GPIN, RARα positively regulates Fmr1 expression via positive regulation of RARγ expression, suggesting that RA induced neural differentiation could maintain Fmr1 expression. On the other hand, Mecp2, the responsible gene of Rett syndrome, negatively related with Fmr1 expression. It is reasonable because Mecp2 plays a role in the transcriptional repression of methylated genes including Fmr1 [20]. However, in TMD-exposed GPIN, Fmr1 was not regulated by RARs, indicating the neural induction by RA was counteracted by TMD. TMD repressed expression of Fmr1 and Mecp2 and MPN analysis also revealed that Fmr1 positively related with Mecp2 in TMD treated EB derivatives. The results mean that TMD repressed expression of Mecp2 via repression of Fmr1 expression. It seemed to contradict the epigenetic silencing of Fmr1 gene by Mecp2. However, Zhang *et al.* reported that Mecp2 mRNA expression level was drastically decreased in the brains of Fmr1 knockout mice, an animal model of fragile X syndrome of autism spectrum [21]. This means the relationship between Fmr1 and Mecp2 is different between normal and pathological neurons. Additionally, Gabrb3, a subunit of GABA A receptor, was positively affected by Mecp2. In Mecp2 deficient mice, subtle dysfunction of GABAergic neurons contributes to numerous neuropsychiatric phenotypes [22]. The relationship of morphological parameters and gene expression parameters was also changed by TMD. RARs became a hub connecting the genes and morphological parameters and NS_formfactor related to expression of some genes independently from other morphological parameters in DMSO control GPIN. This result suggests that RA induced neural differentiation via RARs, thereby, inducing morphological changes. In TMD-exposed GPIN, the morphological parameters were independent from RARs and the expression of Tsc2 related to them via positive connection with NS_formfactor. These results also indicated a counteraction by TMD against the neural induction by RA. Tcs2 is well known to affect cell proliferation and to control cell size and neural development [23]. Therefore, Tsc2 had a high correlativity to morphological parameters.

Parkinson’s disease is the result of degeneration of dopaminergic neuron expressing Th. Recently, some research showed that exposure to pyrethroids including PMT could change the dopaminergic system [24,25]. The genes including in the Parkinson set can be divided into three groups, the ubiquitin pathway (Park2, Snca and Uchl1) and the mitochondrial pathway (Park7, Casp3, Casp7 and Casp9) [26] and genes needed for normal dopaminergic activity (Slc6a3 and Th). In DMSO control GPIN, the ubiquitin pathway genes were not connected into the network. The mitochondrial pathway genes were connected positively but no connection was detected affecting the expression of Th. These results mean that the differentiation of Th positive neuron was not affected by both pathways in normal neuronal differentiation. However, in PMT-exposed GPIN, all genes were connected into the network. Th expression was positively related by Park7, RARβ, Slc6a3 and Uchl1 and negatively related by Snca, Esr1, Crossing_point and NS_formfactor. These results suggest the differentiation of Th positive neuron was affected in a complex manner in PMT exposed EB derivatives. Interestingly, Park7, Casp3, Snca, Park2 and Casp9 were connected indicating the ubiquitin pathway and the mitochondrial pathway affected each other as well as they do in dopaminergic neurons of Parkinson’s disease. Th positive neuron might die by apoptosis because we detected the increased expression of Casp3 and Casp9 in addition to these results. Although all morphological parameters were connected to GPIN in DMSO control, the NS morphological parameters (NS_area, NS_count and NS _perimeter) were not connected to other morphological parameters or genes in the PMT-exposed GPIN. The neurite morphological parameters (Neurite_length, Branch_point, Crossing_point and Posi_area) influenced expression of genes in contrast to the NS morphological parameters. Considering the significant increase of total length of Map2-positive neuron (Figure 2A) and no change in the NS morphological parameters by PMT, the PMT-exposed GPIN successfully drew the change of neuronal morphology.

The comparison of TMD-exposed GPIN or PMT-exposed GPIN with DMSO control GPIN for Autism set and Parkinson’s disease set could be understood without contradicting known pathological pathways. Therefore, we propose that our MPNs approach could draw out the risk of chemicals. The gene expression profiling data of our study have been published on the Profiles of Chemical Effects on Cells (pCEC) system [27], which is a toxicogenomics database with a toxicoinformatics system for risk evaluation and toxicity prediction of environmental chemicals [28] and produced by the National Institute of Environmental Studies, Japan. The microarray data have also been released on the GEO data base [29].

### 2.3. Classification of Chemicals Based on the Values of the Parameters of the Comprehensive Networks

**Figure 5:**
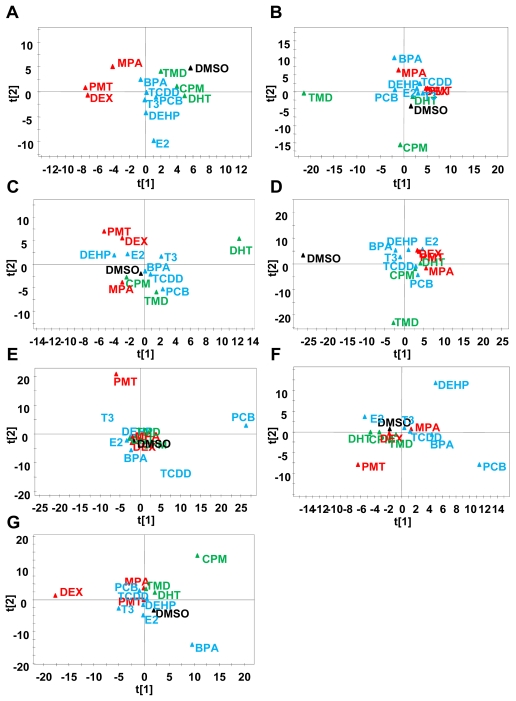
PCA based on Bayesian network parameters. PCA were applied to the Bayesian network parameters based on phenotypic and global gene expression profiling to evaluate the neurotoxicity of 12 environmental chemicals. Score plots based on (**A**) Alzheimer’s disease related gene set; (**B**) Autism related gene set; (**C**) Parkinson’s disease related gene set; (**D**) Axon guidance related gene set; (**E**) Pluripotent related gene set; (**F**) Neural development related gene set; and (**G**) Oxidative stress related gene set.

The genomic data and cell morphological data were converted to the same matrix vector and were used to analyze GPIN. Principal component analysis (PCA) based on the probabilistic relationship data of the GPIN showed that all variance between the 12 chemicals could be described using the first and second principal components (PCs) (Figure 5). The two dimensional PCA plot showed four different groups: DMSO control (black), TMD group (CPM and DHT, green), BPA group [2,3,7,8-tetrachlorodibenzo-*p*-dioxin (TCDD), PCB, T3, bis(2-ethylhexyl) phthalate (DEHP) and E2, blue] and MPA group (PMT and DEX, red) were derived for the Alzheimer’s disease related gene set. The same color coding was used for other experiments, which enabled us to visually recognize changes to the grouping of chemicals. When the largest variable variation was placed in the vertical axis (PC1) and the second variation in the horizontal axis (PC2), the two-dimensional plot showed the position of each chemical. PMT and DEX were located near, but separated from, DMSO in Alzheimer set and Parkinson set. The toxic effects of DEX were reported in animal model of Alzheimer’ disease [30] and Parkinson’ disease [31] although we found no report about PMT in Alzheimer’s disease. In Alzheimer set, E2 was located further away from DMSO than DHT and the opposite positioning was detected in Parkinson set. It might reflect the sexual differences of the diseases as the risk of Alzheimer’s disease is higher in females [32] and that of Parkinson’s disease is higher in males [33]. Because the responsible genes of gender specific Autism spectrum were involved in the Autism set, such gender dependent differences might not be detected in present data. In Autism set, TMD was more separate from DMSO than the others. Indeed other than TMD, the chemicals show no evidence of involvement in autism at present. In the Axon guidance set, all chemicals were almost equally distant from DMSO. As shown in (Figure 2A), all chemicals influenced the total length of Map2-positive neuron at high dose. Therefore, this result is reasonable. In the pluripotent set, PMT and PCB were separated from the others indicating that these chemicals affected the differentiation from ES cells. In fact, PMT and PCB are also located away from DMSO in neural development set. The characterization of chemicals according to their neurotoxic potential reveals that the method described in this current study—that the MPN analysis based on phenotype and gene expression profiling during neuronal differentiation of mESCs—can provide a useful tool to monitor fetal programming and to predict developmentally neurotoxic compounds.

### 2.4. Discussion for Future Work

ESCs test combined with transcriptomics for the assessment of development toxicity has been well studied in recent years [8,34]. However, studies based on the genotype-phenotype profiling are rare. Cell phenotypes are complex and difficult to quantify in a high throughput fashion. The lack of comprehensive phenotype data can prevent or distort genotype-phenotype profiling. Our study described a unique approach to perform multiple phenotype profiling using gene expression data from the early stage of mESC differentiation and morphological data of neuronal cell differentiation after EB formation. Our method provided numerous advantages: (i) Our method can predict multiple phenotype profiles, which could help researchers to reveal different aspects of complex diseases and facilitate treatment design; (ii) Our method can provide a quantitative phenotype description of the sample characteristics; (iii) Our method can extrapolate the profiling to classes beyond those represented in the training data. This is an advantage over traditional classification methods. In contrast, traditional regression methods cannot be directly applied to microarray datasets from different platforms and cannot predict relationships between early events and late phenomena during the differentiation of ES cells into neuronal cells. However, our method can be applied to other types of genomics data such as proteomics or metabolomics. The present study focuses on linear gene-phenotype associations, but more complex relationships can also be devised depending on the data characteristics. Our multi-parametric profiling method for constructing interfering networks of the gene expression data and cellular phenotypic data is only one of many possible approaches. As mentioned above, our MPN analysis can predict the correlation coefficient for each pair of nodes, regardless of the data types. Therefore, our informatics approach and experimental design is also an efficient tool for data integration, mining and network analysis for the other model systems. However, another important issue for the future will be the validation of a larger set of chemicals at a broad concentration range to identify the specific and mechanistically defined markers for differential environmental chemicals.

ES cell-based assays are a promising platform to assess developmental toxicity, because they are capable of recapitulating many of the differentiation states and rely on signaling pathways present in development. We used a neuronal differentiation assay of mESC to assess the activity of groups of environmental chemicals, most of which have *in vivo* toxicity data. The results of this study demonstrated that a subset of tested chemicals are effective in this assay, and that as a statistical analysis, BNA, identified predictive models of detecting fetal programming in the mESC differentiation for a subset of the tested chemicals. Chandler *et al.* demonstrated evaluation of environmental chemicals using a mESC adherent cell differentiation and cytotoxicity assay, showing that genes involved in reactive oxygen species signaling pathways were strongly associated with decreased ES cell differentiation [35]. However, their approaches are linear regression or categorical approaches and are not identical with our approaches. Our approach is unique in linking early gene expression events to the later cellular phenotype features by BNA, which has become popular among biological scientists [36]. Many studies using BNA focus on basic physiological and developmental phenomenon based on cell proliferation [37]. In contrast, our study targets effects of early exposure on late-onset phenotypes, in accordance with the principles of fetal programming against environmental chemicals. In this regard, this is the first study to combine gene expression data and morphological data to estimate the mechanistic path of the response during the early embryonic period.

## 3. Experimental Section

### 3.1. Selection of Test Chemicals

Twelve chemicals, mostly well-characterized medical drugs, pesticides or plastic materials, which have been previously tested by traditional *in vivo* toxicology methods, were used in this study (Table 1). T3, DEX, E2, DHT and MPA are the agonists of the nuclear receptors, ThRs, GR, ERs, AR and RXRs respectively and regulate expression of target genes of each receptor. TCDD also is the agonist of a transcription factor termed AhR [38]. Therefore, these chemicals influence differentiation and development many tissues including neural tissues. CPM, a well characterized teratogen, is the inhibitor of sonic hedgehog (Shh) signal [39]. It can inhibit the acquisition of ventral identity in mESCs-derived neural stem cells [40]. TMD is also well known teratogen of human but not rodents although the toxicological mechanism remains to be unclear [41]. Human epidemiological studies suggested the involvement of TMD in the appearance of autism [42,43]. The studies using rats showed that prenatal exposure to TMD could cause autism-like symptoms in rodents [44]. Prenatal or postnatal exposure to PCB showed long term effects on brain development and behavior in rat [45]. PMT, BPA and DEHP have also shown neurotoxicity in animal models [46–48]. Recently, the TestSmart DNT II meeting to discuss about development of alternative testing methods and models for DNT showed a list of the candidate chemicals for positive control in DNT [5]. 4 chemicals of our list, TMD, PCB, PMT and DEHP are involved in the list. Therefore, the choice of chemicals in present study can be adequate.

### 3.2. Design of Multi-Parametric Profiling Networks Analysis for Detecting Developmental Neuronal Toxicity of Chemicals That Effects Fetal Programming

To evaluate developmental neurotoxicity of these chemicals, we designed a MPN analysis based on gene expression and cellular phenotypic data. The process of MPN analysis was composed of 5 steps (Figure 1). Step 1 involves the exposure of mESCs to chemicals and then the differentiation of mESCs into neuronal cells. Cells were exposed to chemicals for 2 days during Day 0 to Day 2 when initial EBs were formed. Gene expression determination using microarray analysis was performed on RNAs that were sampled immediately after cells were exposed to chemicals. EBs of Day 8 were transferred to poly-dl-ornithine/laminine-coated 24 wells plate and cultured until Day 20 when cells had adequately differentiated to neuronal phenotypes. Differentiated neuronal cells were visualized by immunofluorescence staining. Cell images were acquired automatically using a 10× objective. Gene expression sets selected from microarray data and morphological data of neuronal cells were collected into the same matrix (Step 2). Seven gene expression signatures (pluripotent, neural development, axon guidance, autism, Parkinson’s disease, Alzheimer’s disease and oxidative stress) of biological events and neuronal disease were selected manually and are shown in Table 2. The genes in autism set were chosen based on some reviews [49–51]. The gene in pluripotent set were chosen based on Wang *et al.* [52] and Müller *et al.* [53] The KEGG pathway database was referred to choose genes in other sets. Sex steroid receptors (ESR1, ESR2 and AR) and retinoic acid receptors (RARα, RARβ and RARγ) were added into the autism set, Parkinson set, Alzheimer set to consider the gender depending differences and to consider the effects of neuronal induction by RA *in vitro*, respectively. Once transition matrices were made from gene expression and neuronal cell phenotypes, phenotypic networks and MPNs were derived by BNA. Namely, nodes in the generated GPIN included each of the genes contained in the gene list or each of the morphologic parameters, such as neural cell count or neurite length (Step 3 and 4). We then applied PCA to classify the generated MPN for 7 gene-signature sets of each test-chemical. The values of linkage probability between two nodes in the MPN were used as the parameters in PCA (Step5).

### 3.3. mESC Culture and Maintenance

mESC (B6G-2) derived from Green mouse FM131, a mouse constantly expressing GFP, were cultured on deactivated mouse fibroblast cells (RIKEN, Japan). The proliferated cells were replated on 0.1% gelatin coated dishes with DMEM (phenol red free, Invitrogen, Carlsbad, CA, USA) containing 15% FBS (fetal bovine serum, Invitrogen), 100 μM NEAA (Non-essential amino acids, Invitrogen), 100 μM 2-ME (2-mercaptoethanol, Invitrogen) and 1000 U/mL LIF (Leukemia inhibitory factor, ESGRO, Invitrogen).

### 3.4. EB Formation from mESC and Chemical Treatment

The microsphere array used in this study is a frame separated type (chip 300, STEM Biomethod Corporation, Kitakyushu, Japan), which is made of acrylic resin and the surface has been coated with PDMS resin that is not structured for direct cell adhesion. 1024 wells (diameter 300 μm) were arranged on the surface of the microsphere array. EB formation was performed in the three dimension culture based on the microsphere array. After removal of mouse fibroblast cells, aggregated ES cells were counted and 250 μL cell suspension solution (2 × 10^5^ cells) were put on the microsphere array. Six hours later, the medium was exchanged for each chemical containing medium and incubation continued for 48 h. After that, RNA was isolated for gene expression analysis and culture medium was exchanged for EB medium with add 10 nM retinoic acid for the further morphological analysis. EBs were cultured for 6 days with EB medium replaced every two days. Eight days after chemical exposure, aggregated EBs were replated on Ornithine/Laminine coated 24 wells plate (83 EBs/well). Twenty-four hours later, EB medium was exchanged for neural differentiation medium (DMEM/F12 (1:1), N2 (×100), and 10 ng/mL bFGF) and EBs were cultured for another 20 days, exchanging the medium every 3 days. DMSO was used as the primary solvent for all chemicals, and the DMSO solutions were further diluted in cell culture media for treatments. The final concentrations of DMSO in the media did not exceed 0.1% (vol/vol). The concentrations of chemicals used in this study were: 1 pM and 100 pM for BPA; 1 nM and 10 nM for T3, DEX, E2, DHT, PCB and TCDD; 0.1 μM and 10 μM for CPM, PMT and TMD; 1 μM and 100 μM for MPA and DEHP. The neuronal differentiation parallel to development *in vivo* was confirmed by quantitative RT-PCR of stage specific markers, Oct3, Nanog, Pax6 and Map2 (data not shown).

### 3.5. Immunofluorescence

On Day 20, EBs and differentiated cells were immunostained with Mouse anti-MAP2 antibody (1:200 dilution; Sigma-Aldrich, St. Louis, MO, USA), Mouse anti-GFAP monoclonal antibody (1:200 dilution; Chemicon, GA, USA) and Hoechst 33342 solution (Dojindo, Tokyo, Japan). In brief, cells were fixed with 4% PFA in PBS for 15 min and then blocked for 30 min in PBT buffer (PBS with 5% Goat serum and 0.1% Triton). Cells with primary antibodies were incubated overnight at 4 °C. Cells were washed and blocked in BBT-BSA and then incubated with Alexa-conjugated secondary antibodies (1:1000 dilution, Alexa Fluor 546, Invitrogen). Hoechst 33342 staining was used for counter staining.

### 3.6. Morphological Analysis of mESC, EB and Neuronal Cell Lineages

The immunofluorescence images were acquired using the IN Cell Analyzer 1000 (GE Healthcare, Buckinghamshire, UK). Each neural cell image was analyzed using image analysis software IN Cell Developer Tool Box 1.7 (GE Healthcare). The following 10 parameters were measured: number of all cells (Nuc_count), nucleus area (Nuc_area), the number, area, perimeter and formation of neurospheres (NS), (NS_count, NS_area, NS_perimeter and NS_formfactor), and the shape of nerve cells and the size of neural marker positive cells (posi_area, Neurite_length, Branch_point and Crossing_point).

### 3.7. Gene Expression Analysis and Creation of Candidate Gene Sets

Total RNA on Day 2 of cells derived from mESCs were applied to Illumina beads array systems with the Illumina Mouse WG-6 v1.0 expression beadchip (Illumina, San Diego, CA, USA). The amounts, purity and integrity of RNA were evaluated by UV spectrophotometry and an Agilent Bioanalyzer 2100 (Agilent Technologies, Palo Alto, CA, USA). Genes were normalized with analytical software GeneSpring GX10.02 (Agilent Technologies) [54]. 7 sets of genes were created with reference to the literature to assess the impact on neural development. These categories were Pluripotent, Neural development, Axon guidance, Autism, Parkinson’s disease, Alzheimer’s disease, and Oxidative stress.

### 3.8. Gene and Morphology Interaction Network Analysis

GPIN was quantified to calculate the posterior probability distribution for the strength of the linkages based on gene expression, morphological and chemical exposure dose datasets. Briefly, a GPIN consists of a collection of P nodes, denoted G_1_, G_2_, … G_P_, with observed values n_1_, n_2_, …. n_p_. Define ij (i,j = 1,2, …, P) as parameters in the log-linear function form describing the linkage from node i to node j. Mathematically, this is written as:

(1) $$E[\text{log}(G_{j})]=\sum_{i=1,\neq j}^{P}I_{ij}\beta_{ij}\;\;\text{log}(g_{i})$$

where E[log(G_j_)] represents the expectation for the natural logarithm of G_j_ and I_ij_ (i,j = 1,2, …, P) is an indicator function that equals 1 if node G_i_ has a link to node G_j_, otherwise it equals 0. If a node has a regulatory effect on node G_i_, then that node is referred to as a “Parent of node G_i_” and we refer to it as belonging to the set Pa(G_i_). The prior distribution for the variance is assumed to be inverse Gamma and assuming that the natural log of G_j_ follows a normal distribution with mean and standard deviation, posterior distributions for each parameter can be estimated. The posterior distributions for the linkages were derived using Gibbs sampling. Gibbs sampling has no limitation on the number of possible parents and is easy to cooperate with knowledge information or past experimental results by taking the information into the prior distribution. The goal of the method is to examine the posterior distribution of the linkages between genes. In this study, we applied 20 sets of gene expression data (*N* = 30) and morphological data (*N* = 162). Network was used to evaluate the ability of the algorithm to have higher posterior probability (*P*-value) at the correct linkage in GPIN. In each simulation, Gibbs sampling was performed between 33,000 and 48,000 times. The initial Gibbs sampling was considered to be the burn-in period and was removed in estimating and the last 18,000 to 26,000 iterations were used to establish. *P*-value threshold was set to between 0.995 and 1.0 for up-regulation, 0.47 and 1.0 for down-regulation. Three categories were classified out of the12 GPs depending on network structures.

Class 1: Thick and elongated neurons, but with a small amount of neurite branching. Class 1 could be distinguished from other classes in terms of loading the “Neurite_length” parameter on the top of the PN, such that “Neurite_length” controlled “Branch_point” and “Crossing_point”. The node located towards the bottom seems to suppress neurite growth. The neurite becomes a parent node, which dominates all the other parameters in the PN in order to facilitate its own growth. Namely, the branching points and intersections are increased in parallel with neurite elongation. The parameters of “EB_Area”, “EB_Perimeter” and “EB_FormFactor” are also related to “Neurite_length”, which perhaps suggests that neurites have differentiated normally from EBs and that the shape of NSs is not a circle (*i.e.*, NS becomes flattened during differentiation).

Class 2: Neurite elongation and branching are extensive. In this case, “Branch_point” is located on the top, suggesting that the “Branch_point” controls “Neurite_length” and “Crossing_point”. “Neurite_length” is expressed as the total length of all neurites per cell. “Branch_point” becomes the parent node in this PN because there are many random short neurites and the total length of all the branching short neurites at their branch points is regarded as the neurite length. Therefore, the promotion arrow from the branch point tends to be the parameter of neurites. Because there are so many random branch points, it is very likely that there are many short crossing intersections. Furthermore, since there are so many branches from the neurites which perhaps did not differentiate from EBs, the parameter of “Branch_point” might not be related to EB shape. Consequently, the EB shape tends to be round compared with that of Class 1 EBs.

Class 3: larger NSs and less neurites. Different from classes 1 and 2, “Nuc_count” and “Nuc_area” are localized at the top in this PN. This suggests that cell proliferation in NSs is more predominant than neural cell outward migration. Common to these three classes, in case of that differentiated neural cell expanded outside of EB and neural differentiation related morphological parameters emerged above of PN. These parameters exert influence on the number of cells and the shape of the EB. Furthermore, when the differentiation is advanced, the PN tends to become complex. In fact, neural differentiation is not too advanced like as Class 3, it became the result of locating the parameter related to number of cells in the high rank from the parameter of the neuronal cell. The parameter concerning the EB is always located in the subordinate position of the PN on any PN and this tendency corresponded to the theory that the shape changed depending on the number of cells and the progression of neuronal differentiation.

### 3.9. Statistical Analysis

All experiments in this study were performed in triplicate to test the reproducibility of the results. Statistical analysis was performed by two-tailed Student’s *t*-test. Relationships were considered statistically significant with *p* < 0.05.

## 4. Conclusions

Our study provides an advanced framework to integrate the gene expression and neuronal cell phenotypes for target prediction. Thus a combination of BNA and PCA clustering could provide compound-target prediction efficiency. We believe this method has considerable potential. For example, new markers could be implemented that enable predictive toxicology of active lead compounds. Combined with chemical structure knowledge and ligand-target prediction, such approaches could provide detailed mechanistic insight to help guide medicinal chemists early in the lead optimization process. Dealing with complexities of predictive toxicology will require breakthroughs in cellular image analysis, target prediction schemes and data mining. Our integration analysis of cellular phenotypes with gene expression represents a step forward in solving the DNT for environmental chemical assessment.

## Figures and Tables

**Table 1:** Summary of 12 test chemicals.

Chemical Name	Ellipsis	Intended Use	Physiological Effect and Toxicity	Mode of Action	Target Protein
Triiodotyronine	T3	Endogenenous hormne	Pseudo thyroid hormone	transcriptional regulation	Thyroid hormone receptor (TR)α, TRβ
Dexamethazone	DEX	Medicinal drug	Pseudo corticosteroid hormone	transcriptional regulation	Glucocorticoid receptor (GR)
17b-Estradiol	E2	Endogenenous hormne		transcriptional regulation	Estrogen receptor (ER)α, ERβ
5a-Dihydrotestosterone	DHT	Endogenenous hormne		transcriptional regulation	Androgen receptor (AR)
2,3,7,8-tetrachlorodibenzo-*p*-dioxin	TCDD	Unintentional chemical	Multi-toxicity	transcriptional regulation	Aryl hydrocarbon receptor (AhR)
Methoprene acid	MPA	Pesticides	Teretogenecity	transcriptional regulation	Retinoid X receptor (RXR)α, RXRβ, RXRγ
Cyclopamine	CPM	Medicinal drug	Teretogenecity	Signal inhibition	Hadgehog signaling pathway
Thalidmide	TMD	Medicinal drug	Teretogenecity and Autism	Unknown	Oxidative stress
4(OH)-2′,3,3′,4′,5′-pentachlorobephenyl 107	PCB	Metabolite of PBC	Multi-toxicity	Unknown	Unknown (ERα, oxidativestress)
Permethrin	PMT	Pesticides	Neuro-toxicity	Unknown	Oxidative stress
Bisphenol A	BPA	Plastic materials	Reproductive and Neuro-toxicity?	Unknown	Unknown (ERα, ERRγ)
Bis(2-ethylhexyl) phthalate	DEHP	Plastic materials	Reproductive and Neuro-toxicity?	Unknown	Unknown [Peroxisome proliferator-activated receptor (PPAR)α, antiTR]

**Table 2:** Lists of 7 gene sets selected for network analysis.

Alzheimer	Autism	Parkinson	Axon Guidance	Pluripotent	Neural Development	Oxidative-Stress
AR	AR	AR	1500003O03Rik	Arid3b	Atbf1	Aass
ApoE	Cntnap2	Casp3	Abl1	Esrrb	Cdyl	Als2
App	En2	Casp7	Ablim1	Fkbp3	Fos	Apoe
Bace	Esr1	Casp9	Cfl1	Hdac2	Gbx2	Ctsb
Casp3	Esr2	Esr1	Cxcl12	Klf4	Gfap	Dnm2
Casp7	Fmr1	Esr2	Efna4	Mybbp1a	Hras1	Fancc
Esr1	Foxp2	Park2	Epha2	Nacc1	Map2	Gpx7
Esr2	Gabrb3	Park7	Ephb1	Nanog	Mapk1	Gpx8
Ide	Mecp2	RARa	Nfatc2	Nfkbib	Mapk3	Gusb
Il1r1	Nlgn3	RARb	Nfatc3	Nr0b1	Nestin	Hprt1
Mme	RARa	RARg	Ntng1	Nr5a2	Pla2g6	Kif9
Psen	RARb	Slc6a3	Sema3a	Pou5f1	Raf1	Noxo1
RARa	RARg	Snca	Sema3b	Rex1	Rhog	Nxn
RARb	Reln	Th	Sema3d	Sall4	Rif1	Park7
RARg	Slc6a4	Uchl1	Sema3f	Smarcad1	Rps6ka1	Ppp1r15b
Tnfrsf1a	Tsc1		Sema3g	Smarcc1	Sall1	Prdx2
	Tsc2		Sema6a	Sox2	Shc1	Prdx6-rs1
	Ube3a		Sema6b	Sp1	Smarcad1	Psmb5
			Sema6d	Spag1	Sox2	Recql4
			Srgap3	Trim28	Tuj1	Scd1
			Unc5d	Zfp281	Map2k1	Slc41a3
				*c*-Myc		Sod1
						Sod3
						Txnip
						Txnrd1
						Xpa
